# Characterization of Loss-Of-Function *KCNJ2* Mutations in Atypical Andersen Tawil Syndrome

**DOI:** 10.3389/fgene.2021.773177

**Published:** 2021-11-25

**Authors:** Pauline Le Tanno, Mathilde Folacci, Jean Revilloud, Laurence Faivre, Gabriel Laurent, Lucile Pinson, Pascal Amedro, Gilles Millat, Alexandre Janin, Michel Vivaudou, Nathalie Roux-Buisson, Julien Fauré

**Affiliations:** ^1^ Université Grenoble Alpes, Inserm, U1216, CHU Grenoble Alpes, Grenoble Institut Neurosciences, Grenoble, France; ^2^ CEA, CNRS, Institut de Biologie Structurale, Université Grenoble Alpes, Grenoble, France; ^3^ Medical Genetics Department, Dijon Bourgogne University Hospital, François Mitterand Hospital, Dijon, France; ^4^ Cardiology Department, Dijon Bourgogne University Hospital, François Mitterand Hospital, Dijon, France; ^5^ Medical Genetics Department, University Hospital, Montpellier, France; ^6^ Département de Génétique Médicale, Maladies Rares et Médecine Personnalisée, Montpellier, France; ^7^ Genetic Department for Rare Diseases and Personalized Medicine, Clinical Division, Montpellier, France; ^8^ Pediatric and Congenital Cardiology Department, Clinical Investigation Centre, PhyMedExp, CNRS, INSERM, University of Montpellier, University Hospital, Montpellier, France; ^9^ Laboratoire de Cardiogénétique Moléculaire, Centre de Biologie et Pathologie Est, Hospices Civils de Lyon, Lyon, France

**Keywords:** catecholaminergic polymorphic ventricular tachycardia, Andersen-Tawil syndrome, Kir2.1 channel, Pierre Robin sequence, functionnal characterization, KCNJ2 variants

## Abstract

Andersen-Tawil Syndrome (ATS) is a rare disease defined by the association of cardiac arrhythmias, periodic paralysis and dysmorphic features, and is caused by *KCNJ2* loss-of-function mutations. However, when extracardiac symptoms are atypical or absent, the patient can be diagnosed with Catecholaminergic Polymorphic Ventricular Tachycardia (CPVT), a rare arrhythmia at high risk of sudden death, mostly due to *RYR2* mutations. The identification of *KCNJ2* variants in CPVT suspicion is very rare but important because beta blockers, the cornerstone of CPVT therapy, could be less efficient. We report here the cases of two patients addressed for CPVT-like phenotypes. Genetic investigations led to the identification of p. Arg82Trp and p. Pro186Gln *de novo* variants in the *KCNJ2* gene*.* Functional studies showed that both variants forms of Kir2.1 monomers act as dominant negative and drastically reduced the activity of the tetrameric channel. We characterize here a new pathogenic variant (p.Pro186Gln) of *KCNJ2* gene and highlight the interest of accurate cardiologic evaluation and of attention to extracardiac signs to distinguish CPVT from atypical ATS, and guide therapeutic decisions. We also confirm that the *KCNJ2* gene must be investigated during CPVT molecular analysis.

## Introduction

Andersen Tawil Syndrome (ATS) is characterized by a triad of symptoms: periodic paralysis (episodic flaccid muscle weakness), distinctive clinical features (such as low-set ears, broad forehead, small palpebral fissures, hypertelorism, broad nasal root, bulbous nose, thin upper lip, maxillary, malar and mandibular hypoplasia, abnormal extremities), and cardiac arrhythmias. Less frequently, skeletal, dental and renal findings are present (Andersen, 1971, Tawil, 1994, Andelfinger et al., 2002; Tristani-Firouzi et al., 2002).

Pathogenic variants in *KCNJ2* were first associated with Andersen Tawil Syndrome (ATS) in 2001 (Plaster et al., 2001) and until now account for 80% of the cases (Zhang et al., 2005; Haruna et al., 2007). *KCNJ2* encodes the pore-forming alpha subunit of the potassium channel Kir 2.1 that underlies the inward rectifier potassium current IK1stabilizing resting potential during cardiac and skeletal myocytes repolarization (Plaster, et al., 2001; Wible et al., 1995; Sumitomo, 2016). *KCNJ2* variants are also associated in rare familial forms of atrial fibrillation type 9, short QT syndrome type 3 and Catecholaminergic Polymorphic Ventricular Tachycardia (CPVT) (Tester et al., 2006).

CPVT is a heritable arrhythmia syndrome characterized by symptoms of palpitations, dizziness, syncope or sudden death triggered by physical activity or sympathetic nervous system stimulation such as acute emotion or stress (Hayashi et al., 2009; Ackerman et al., 2011). While resting ECG is usually normal, exercise stress test may unmask ventricular arrhythmias (VA), arising in the absence of structural cardiac abnormalities. VA range from premature beats to sustained ventricular tachycardia (VT) as workload increases, the most pathognomonic aspect observed being bidirectional VT (BVT) in 1/3 cases (Andersen 1971). In 50–65% of CPVT cases genetic mutations are identified in *RYR2*, a gene encoding the main intracellular calcium channel of cardiomyocytes, and much less frequently in others genes that are also related to calcium homeostasis [*CASQ2*, *CALM1, CALM2, CALM3, TRDN, TECRL* (Priori et al., 2002; Ackerman et al., 2011; Ackerman et al., 2011; Nyegaard et al., 2012; Roux-Buisson et al., 2012; Devalla et al., 2016; Jensen et al., 2018)]. Phenocopies of CPVT have been described in association with mutations in *KCNJ2* but to date, only a few mutations of *KCNJ2* were clearly showed responsible for a CPVT phenotype. In the case of atypical arrhythmia, ATS or CPVT diagnosis may therefore both be evoked. In such cases the identification of the mutated gene is of uppermost importance because first line treatment of *RYR2* mutation-induced CPVT is based on beta blockers that can be less efficient on *KCNJ2* mutated cases (Hayashi et al., 2009; Priori et al., 2013; Manzatti et al. 2020).

We report here two cases of atypical ATS initially addressed for suspicion of CPVT for which mutations in *KCNJ2* were identified. Molecular characterization of the mutations allowed to clarify the pathophysiology of the two cases and add a new pathogenic *KCNJ2* variant in databases. Our report also adds a new description of CPVT patients with *KCNJ2* mutations, and specific features of such patients are discussed.

## Methods

### Ethics Statement

All clinical investigations reported in this study were realized for medical purpose. Molecular genetics analysis was performed after obtaining written informed consent of the patients and/or their legal guardians for children. Written authorization for publication was obtained from every individual and/or their parent/guardian.

Animal handling and experiments fully conformed with European regulations and were approved by the Ethics Committee of the Commissariat à l’Energie Atomique et aux Energies Alternatives (Ethics Approval #12-040). Authorization of the animal facility has been delivered by the regional administration (Préfet de l’Isère, authorization # D 38,185 10,001).

### Patients

Probands and relatives were referred at the University Hospital of Dijon, France and the University Hospital of Montpellier, France for the families 1 and 2 respectively. Clinical data, resting electrocardiogram, exercise stress test, 24 h Holter monitoring, transthoracic echocardiography and cardiac magnetic resonance (CMR) imaging were performed on each proband. Proband 1 also underwent coronary computed tomography angiography (CCTA).

### Genetic Analysis

Genomic DNA was extracted from blood’s leucocytes with an automated technique using the Blood XL kit (Macherey Nalgel). For each proband addressed for CPVT, the analysis of the coding sequence and 10 base pairs of the intron-exon junction for *RYR2* (NM_001035.2), *CASQ2* (NM_001232.3), *TRDN* (NM_001256021), *CALM1* (NM_006888.4) and *KCNJ2* (NM_000891.2) genes were sequenced with Ion Torrent PGM sequencer (Thermo Fisher). Bioinformatics analyses were performed using the Torrent_Suite 5.0, Torrent variant calling v5.0.4. Variants were annotated using two independent softwares, Ion Reporter v5.0.4.0 and a local plug-in. Exon not covered by the gene panel at a minimal depth of 30X and variation of class 3, 4 or 5 according to ACMG recommendations (Richards et al., 2015) were controlled by Sanger sequencing on an ABI 3130xl sequencer (PE Applied Biosystems®, Foster City, United States). Analysis of the chromatograms was performed with SeqScape V (PE Applied Biosystems®, Foster City, United States). The segregation analysis of *KCNJ2* variants within the family was performed by targeted Sanger sequencing when the relative DNAs and clinical data were available.

### Functional Characterization

Human Kir2.1 in the oocyte expression plasmid pXoom was a kind gift from Dr Saïd Bendahhou, from Nice Sophia Antipolis University. The p. Arg82Trp and p. Pro186Gln mutations were inserted by site directed mutagenesis using the QuickChange II Site-Directed Mutagenesis Kit (Agilent, Santa Clara CA, United States). The entire coding sequence was verified by Sanger sequencing.

*Xenopus* oocytes were prepared as previously reported (Moreau et al., 2017; Vivaudou et al., 2017). The protocols used to record and analyze data are detailed in Supplementary Figure S1. Briefly, after surgical retrieval, the oocytes are defolliculated by type 1A collagenase. Each oocyte was injected with 50 nl of RNAse-free water containing 2 or 50 ng of Kir2.1 mRNA. Microinjected oocytes were incubated for more than 2 days at 19°C in Barth’s solution (in mM: 1 KCl, 0.82 MgSO_4_, 88 NaCl, 2.4 NaHCO_3_, 0.41 CaCl_2_, 16 Hepes, pH 7.4) supplemented with 100 U.ml^−1^ of penicillin and streptomycin and 0.1 mg ml^−1^ of gentamycin. Whole-cell currents were recorded with the two-electrode voltage clamp (TEVC) technique using a HiClamp robot (MultiChannels System) (Vivaudou et al., 2017). Microelectrodes were filled with 3 M KCl and oocytes were bathed in solutions all containing in mM: 1.8 CaCl_2_, 1 MgCl_2_, 5 HEPES, 3 niflumic acid to block endogenous Cl^−^ currents (pH 7.4). High K^+^ solution contained also 91 mM KCl, Low K^+^ solution 91 mM NaCl and 2 mM KCL, and Ba^2+^ solution 91 mM KCl and 3 mM BaCl_2_. Currents were recorded at −50 mV while oocytes were bathed sequentially for 10 s in Low K^+^, High K^+^ and Ba^2+^ solutions. Current-Voltage curves were obtained in each solution using a ramp from −50 to +50 mV. These curves were used to verify that the recorded currents were inwardly rectifying as expected from Kir2.1 current. Small outward currents at positive potentials were often observed and are thought to be from endogenous currents because they were not blocked by Ba^2+^. Oocytes displaying large (>1 µA) outward currents at positive potentials were discarded as too leaky. The values shown in the figures are those recorded at −50 mV.

Binomial analysis of the subunit distribution in a tetrameric channel was performed as previously described (Hosy and Vivaudou, 2014). Oocytes display a notorious variability in terms of expression levels from batch to batch. To minimize this source of error when comparing current amplitudes, currents arising from different coexpression ratios were obtained and normalized by considering only the same batches of oocytes. Curve fitting was performed with a modified version of the eeFit program (Vivaudou, 2019).

Average values are presented as mean ± s.e.m.

All detailed protocols are available upon request.

## Results

### Clinical Data of CPVT Probands

**FIGURE 1:**
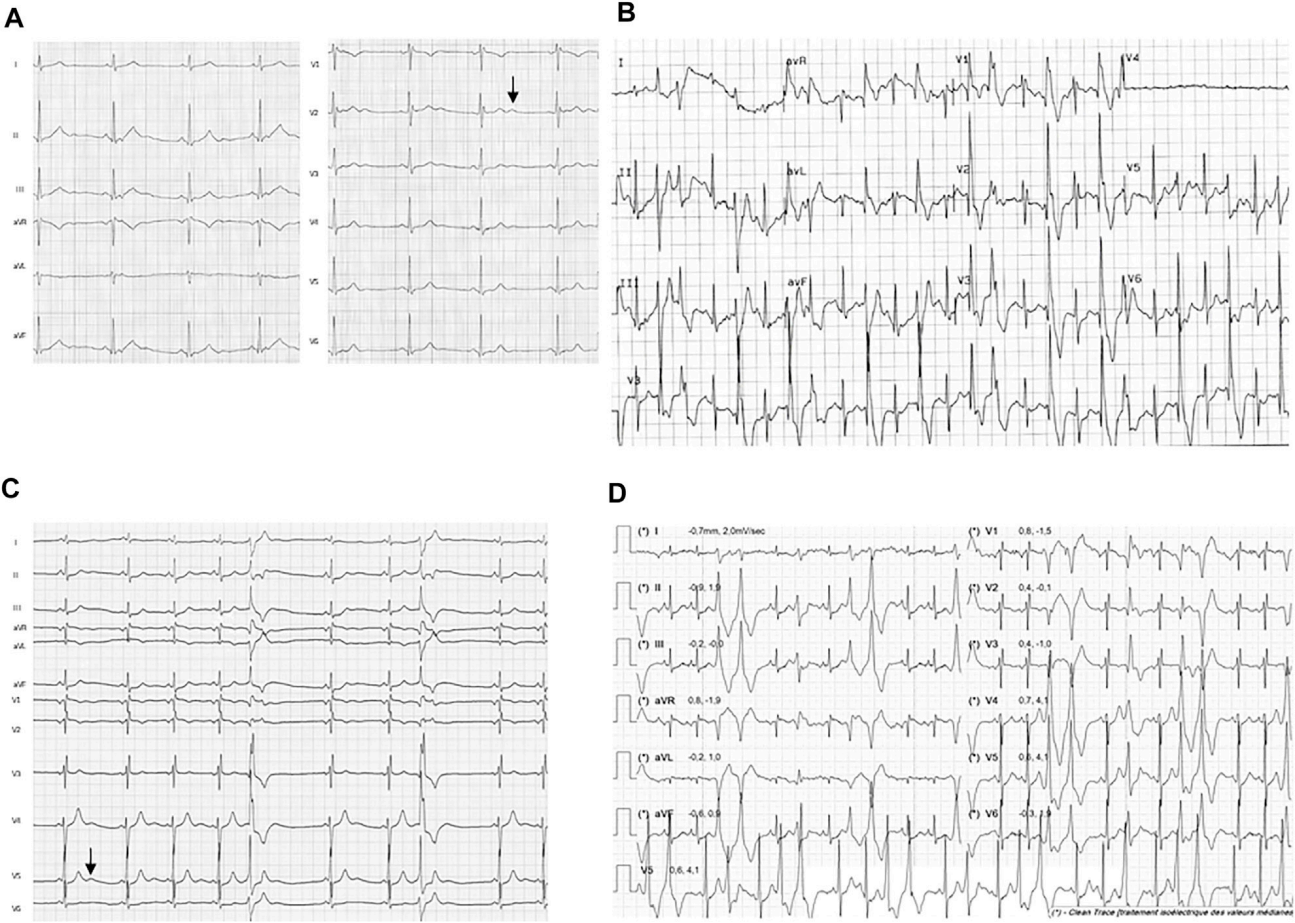
Resting ECG and exercise ECG from the probands 1 and 2. Traces from proband 1 shows **(A)** resting ECG with U wave (arrow) and **(B)** PVCs during exercise test (120 W workload). Traces from proband 2 shows **(C)** resting ECG with U wave (arrow) and **(D)** polymorphic PVCs at exercise (50 W workload) during the first Cardiopulmonary exercise test (CPET) before medical treatment was started.

Patient 1 was a young woman whose first symptoms started at the age of 16. She was referred to the hospital for episodes of palpitations triggered by exercise or emotion, occurring at least once a month. Dizziness with hypotension occurred during physical exercise, without loss of consciousness. With time she developed symptoms of dyspnea, hot flushes, chest pain and asthenia associated with the palpitations. Her resting ECG was normal (QTc of 412 ms) except for asymptomatic monomorphic premature ventricular contractions (PVC) with right bundle branch block (RBBB) pattern. The exercise stress test revealed several RBBB PVCs with left axis, increasing with workload and junctional beating, but no VT (Figures 1A,B). The 24-hour-holter monitor evidenced diurnal PVC mostly isolated with rare doublets, and premature atrial contractions (PAC) spread over the recording. Transthoracic echocardiography showed a moderate dilatation of the right ventricle and CMR did not show other signs. She had no history of periodic paralysis and did not present with any particular morphological or extracardiac features. She was the first daughter of non-consanguineous parents, who were asymptomatic and presented no symptoms upon cardiologic explorations. No significant cardiac event was reported from the family history, other than atrial fibrillation in her paternal grandfather. Altogether, these data lead to a diagnosis of CPVT. Several first-line drugs were administered (propranolol, propafenone, sotalol), with no efficacy. Nadolol was finally introduced and implantable cardioverter defibrillator is under discussion.

Patient 2 was a teenager female who was referred to pediatric cardiology at the age of 13 since asymptomatic arrhythmia was fortuitously detected on resting ECG. She reported suffering from muscular pain and weakness of lower limbs. She was referred to the clinical genetic department where retro-micrognathia, relative short and low set ears and clinodactyly of the fifth digit were observed. Stature was within normal range (150 cm heigh, −1DS for 38 kg weight, −1DS). In clinical history, a cleft palate surgery was found at the age of 6 months as part of a postnatal Pierre Robin Sequence.

On resting ECG, polymorphic single PVCs were present with normal QTc evaluated at 430 ms and U waves (Figure 1C). Exercise test showed an increase with workload in numbers and polymorphic aspects of PVCs, but no sustained VT (Figure 1D). A 24-h Holter monitoring evidenced numerous polymorphic PVCs and few non-sustained VT (Supplementary Figure S2A). A correlation between the number of PVCs and the heart rate was observed (Supplementary Figure S2B). All cardiac anomalies reported on both 24-h Holter monitoring and exercise test were asymptomatic. Transthoracic echocardiography, CMR and CCTA were all normal. The analysis of a gene panel involved in cleft lip and palate failed to find pathogenic mutations and a high-resolution array CGH was normal.

There was no family history of arrhythmias, and transthoracic echocardiography and 24 h-holter ECG were normal on both parents. There was no other significant family history for both probands.

Additional extracardiac features in proband 2 allowed retrospective diagnosis of Andersen Tawil syndrome with CPVT. Beta-blocking therapy, flecainide were poorly efficient or showed adverse effects and potassium supplementation had positive effects.

### Identification of *de novo KCNJ2* Variants

Targeted sequencing was performed for both patients with a panel of genes involved in arrhythmia (*RYR2*, *CASQ2*, *TRDN*, *CALM1* and *KCNJ2*). In proband 1, a *de novo* heterozygous missense variant c.244C > T; p. Arg82Trp of the *KCNJ2* gene was identified (accession number: VCV000067568). DNA from her sister was unfortunately not available. In proband 2, a c.557C > A; p. Pro186Gln heterozygous missense variant in *KCNJ2* was found *de novo* (accession number: VCV000652820). The variant was not found in the DNA of her asymptomatic sister. No variants (classe 3, 4 or 5 ACMG) that could be responsible for the proband symptoms were evidenced in other genes for both probands.

Both variants of *KCNJ2* are non-conservative amino acid substitutions that impact residues highly conserved across species. They were predicted damaging by five softwares (Polyphen2, Provean, CADD, FatHMM, Mutation Taster), and unreported in the GnomAD database (v2.1.1). Both variations may impact important functional domains of the protein: arginine 82 is located in the first transmembrane segment of the protein while proline 186 belongs to a C-terminal PKKR motif involved in the binding of the channel modulator phospholipid phosphatidyl inositol bisphosphate (PIP_2_) (Handklo-Jamal et al., 2020). The p. Arg82Trp variant of *KCNJ2* had previously been associated to CPVT (Tester et al., 2006), and the p. Arg82Gln variant affecting the same amino acid had been reported several times with ATS or ATS with cardiac phenotype alone (Kimura et al., 2012). To our knowledge, the p. Pro186Gln variant has never been reported in the literature and is absent from the HGMDPro and LOVD databases but is reported once in ClinVar database as Likely Pathogenic in a patient presenting with ATS. However, other variants affecting the same codon and leading to different amino acid transition were reported in ATS (p.Pro186Leu, p. Pro186Ala and p. Pro186Thr) (Soom et al., 2001; Song et al., 2016).

### Impact of p.Arg82Trp and p.Pro186Gln Variants on Kir2.1 Function

**FIGURE 2:**
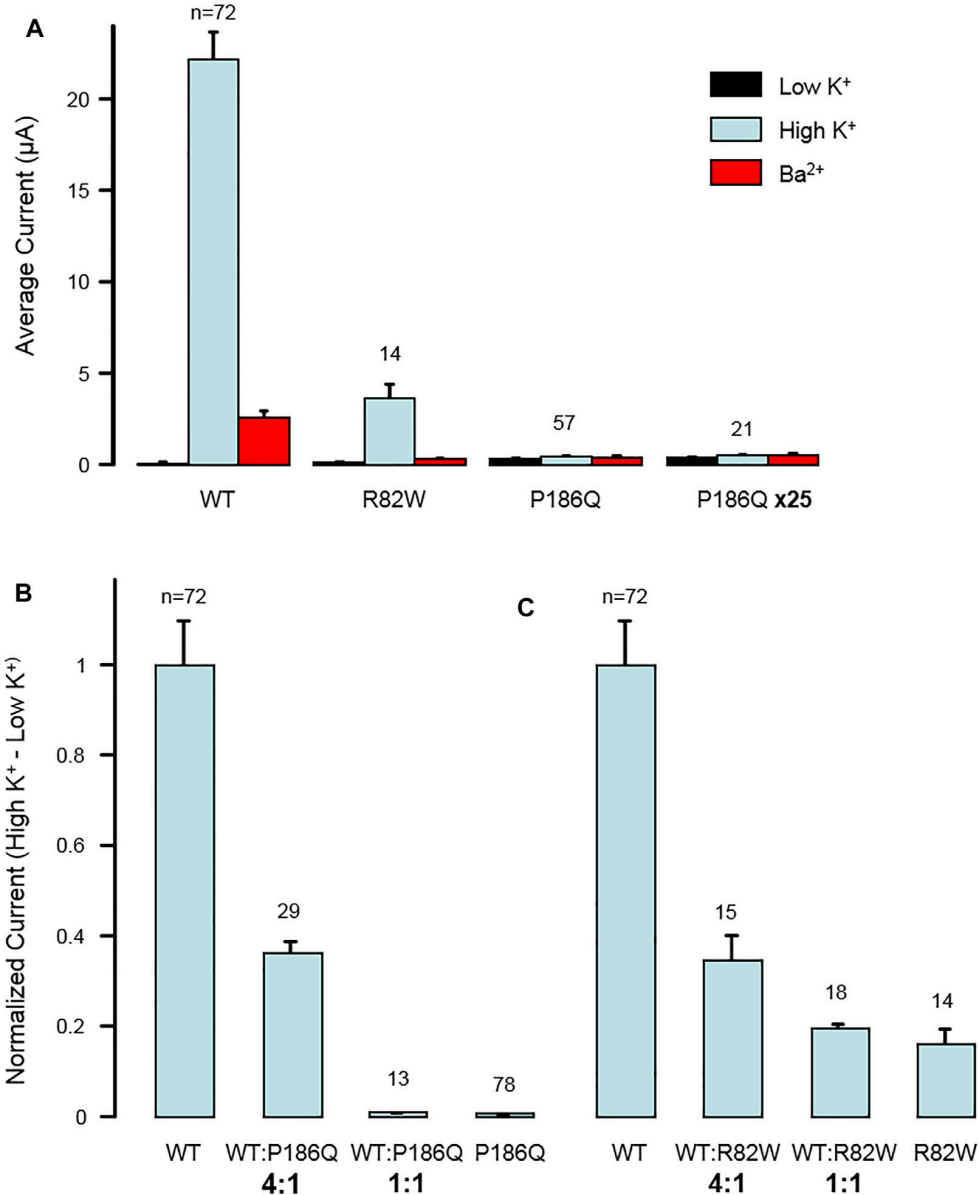
Dominant negative effect of mutations p. Arg82Trp (R82W) and p. Pro186Gln (P186Q) on Kir2.1 function. **(A)** Mutations reduce the activity of homotetrameric channels. The histogram displays the average whole-cell currents recorded in Xenopus oocytes injected with 2 ng of RNA coding for either wild-type Kir2.1 (*WT*), Kir2.1_R82W_ (*R82W*), or Kir2.1_P186Q_ (*P186Q*). Oocytes injected with 50 ng of Kir2.1_P186Q_ were also tested (*P186Q x25*). Measurements were done successively in extracellular solutions containing 2 mM K^+^ (*Low K* ^*+*^), 96 mM K^+^ (*High K* ^*+*^), and 3 mM Ba^2+^ and 96 mM K^+^ (*Ba* ^*2+*^). **(B,C)** Effect of mutations varies gradually with the ratio of WT to mutant subunits. **(B)** The histogram displays the average whole-cell currents recorded in Xenopus oocytes injected with RNAs coding for wild-type Kir2.1 (*WT*) or Kir2.1_P186Q_ (*P186Q*), or with a mixture of RNAs coding for WT and mutant with ratios of 4:1 or 1:1. The currents were calculated as the difference between the current measured in High K^+^ solution and the current measured in Low K^+^ solution. They are here normalized to the currents obtained with only WT subunits. **(C)** Same for mutation R82W. Numbers above bars indicate the number of oocytes included in the averages. Error bars indicate s. e.m.

**FIGURE 3:**
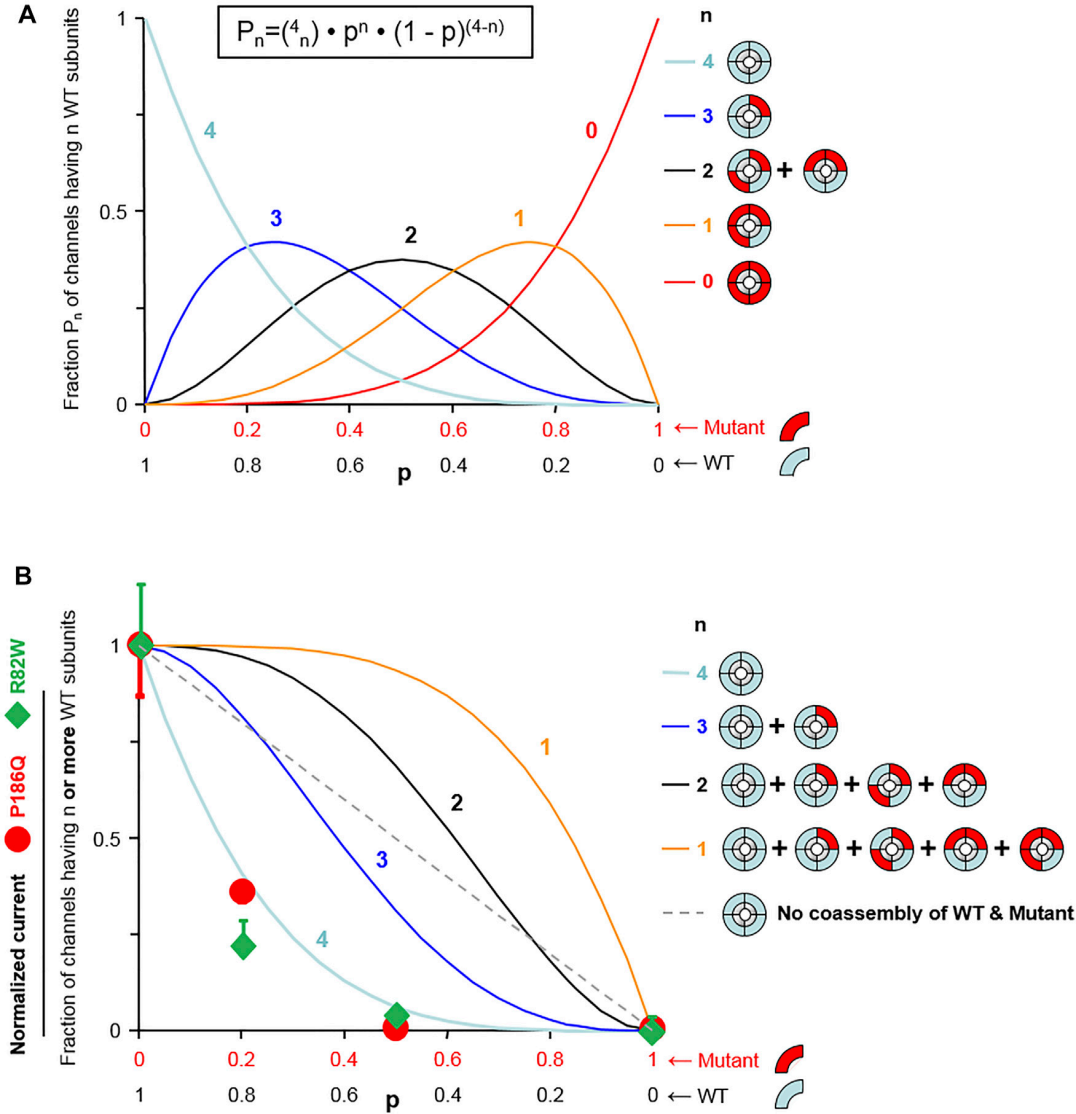
Model fitting reveals that one mutant subunit is sufficient to impair the function of Kir2.1 tetrameric channels. **(A)** Assuming random assembly of WT and mutant subunits, the probability of occurrence of channels having exactly n WT subunits (P_n_) follows a binomial distribution (equation shown at top). That probability P_n_ is shown as a function of the fraction of wild-type subunits (p) for each possible value of n (n indicated next to the curves). **(B)** Probability of a channel having n or more WT subunits calculated using the distributions of **(A)** (n indicated next to the curves). The straight dotted line represents the probability of a WT channel if WT subunits and mutant subunits could not co-assemble. The symbols represent normalized K^+^ current. The percent deviation of the experimental data from each model, calculated from the rmsd (root mean-square deviation) is for p. Pro186Gln: 3.6% (*n* = 4), 28% (*n* ≥ 3), 46% (*n* ≥ 2), 56% (*n* ≥ 1), and 33% (no WT/mutant mixing; dotted straight line); and for R82W: 9.4% (*n* = 4), 33% (*n* ≥ 3), 49% (*n* ≥ 2), 59% (*n* ≥ 1), and 37% (no WT/mutant mixing; dotted straight line). For both mutants, the experimental data match best the model *n* = 4, meaning that channels are fully functional only when they contain no mutant subunits.

The impact of both variants was analyzed by measuring currents generated after expression of Wild Type (WT) or mutated Kir2.1 in *xenopus* oocytes. Examples of recording from an oocyte injected with variable amount of *KCNJ2* mRNA are detailed in Supplementary Figures S3, S4. Whole cell recordings showed that specific Kir2.1 currents detected in the presence of high extracellular potassium concentration, and abolished by the blocking ion barium, were drastically reduced by the p. Arg82Trp mutation. The p. Pro186Gln mutant was not detectable even when oocytes were injected with high RNA amount (Figure 2). These experiments indicated that both mutations induce a partial (p.Arg82Trp) or total (p.Pro186Gln) loss of channel activity that could be due to alterations of channel function, protein expression or localization. The Kir2.1 channel is a homotetramer and we next tested its function upon expression of various ratios of WT and mutants. As described in Figure 3, co-expression of each mutant with WT Kir2.1 in a 1 to 4 ratio induced a 60% decrease in the current, whereas a 1:1 WT/P186Q ratio resulted in a 95% decrease in the current and a 1:1 WT/R82W resulted in 80% reduction. The 1:1 ratio mimics the phenotype expected in a patient having a heterozygous mutation. These results suggest that both mutants of Kir2.1 were expressed and could assemble with the WT protein, generating inefficient heteromultimers. However the p. Pro186Gln mutant could have a more severe effect on the channel. A model showing the number of heterotetramers of the channel including exactly (Figure 3A) or at least (Figure 3B) 1, 2, 3 or 4 WT subunits was produced as a function of the ratio of WT or mutant mRNAs injected in oocytes, with the reasonable assumption that WT and mutant subunits assemble randomly to produce tetrameric channels. The normalized amount of current produced by the various ratios of WT and mutated Kir2.1 obtained in Figure 3 followed the curve representing the proportion of homotetramers of WT Kir2.1 (Figure 3B). This indicates that in co-expression experiments, the detected current may only come from WT homotetramers of Kir2.1, and that the incorporation of a single mutated subunit is sufficient to confer the mutant phenotype to the whole Kir2.1 tetramer. Because the p. Arg82Trp mutant channel retains some activity, this mutation has less drastic adverse effects than the p. Pro186Gln mutation.

Overall we showed that expression of both p. Arg82Trp and p. Pro186Gln mutants in *xenopus* oocytes produced respectively impaired or non-functional Kir2.1 channels. Co-expression of either mutant with WT protein strongly affected the function of the tetrameric channel, showing a dominant negative effect of the mutations. These functional studies correlated with the dominant mode of inheritance of the CPVT phenotypes in the two families, and confirmed the deleterious effect of the genetic variations of *KCNJ2* found in both patients. Considering this, both variants p. Arg82Trp and p. Pro186Gln were classified Pathogenic (class 5) according to ACMG classification (Richards et al., 2015).

## Discussion

We report here two sporadic cases of atypical ATS associated with *KCNJ2* mutations, the recurrent p. Arg82Trp and a so far uncharacterized p. Pro186Gln. Both variants occurred *de novo* in the proband as observed in one third of *KCNJ2* cases (Manzatti et al., 2020). Cellular studies performed after expression in *xenopus* oocytes showed that both variants reduced drastically Kir2.1 channel function.

*KCNJ2* encodes the pore-forming subunits of the tetrameric potassium channel Kir 2.1 that plays a major role in the stabilization of the resting potential during cardiac and skeletal myocytes repolarization (Wible et al., 1995; Plaster et al., 2001). Loss-of-function *KCNJ2* mutations are associated with ATS whereas gain of function mutations are associated with autosomal dominant AF familial 9 and short QT syndrome 3 (Xia 2005 Priori et al., 2005). A total of 147 variants are listed modifying the coding sequence are reported for *KCNJ2* gene in the ClinVar database (missense, nonsense, frameshift). Nine are truncating mutations while the others are missense variations. All, except one, are associated to a cardiac phenotype or to ATS, and forty-two variants considered as probably pathogenic or pathogenic are located on 27 amino acid positions that correspond to hot spots of mutations, including amino acid 82 and 186 that are mutated in our report. Eighty-five variants are classified as variant of unknown significance, what underlines the need of functional study to improve molecular diagnosis for *KCNJ2* variants.

Here, using expression in *xenopus* oocytes, we demonstrated that p. Arg82Trp and the p. Pro186Gln mutations of Kir2.1 reduced drastically the channel activity. Previous data published on the p. Arg82Trp variant showed that expression of the mutant allele alone completely abolished outward and inward currents and that its trafficking to cell membrane was normal (Eckhardt et al., 2007; Kimura et al., 2012). Although all experiments performed with this mutant concluded to a reduction of inward current, the notion that it had a dominant negative effect was more controversial (Eckhardt et al., 2007; Kimura et al., 2012). Our experiments demonstrate that a homotetramer of p. Arg82Trp mutated subunits retain some potassium channel activity, confirming that a least a fraction of the expressed proteins can reach plasma membrane, and the mutation does not fully abolish the function of the channel. In our experiments, the p. Arg82Trp mutant of Kir2.1 also showed a clear dominant negative effect whenever expressed in combination with the WT protein. Of note, another recurrent mutation on the same amino acid, the p. Arg82Gln has also been reported and characterized as having a dominant negative effect (Davies et al., 2005; Kimura et al., 2012).

To our knowledge, the variant p. Pro186Gln of Kir2.1 has never been reported in the literature but is referenced in ClinVar database. Substitutions affecting the same codon and leading to different amino acid variations (p.Pro186Leu and p. Pro186Thr) were however already reported with a dominant negative effect (Soom et al., 2001; Song et al., 2016), and functional studies on the variant p. Pro186Ala demonstrated a significantly reduction of PIP_2_ binding *in vitro* (Soom et al., 2001). Our experiments demonstrating a loss of function of channel due to the p. Pro186Gln mutation confirm therefore the critical role of the Proline 186 residue in Kir2.1, as expected from its position within the PIP_2_ binding site of Kir2 channels (Hansen et al., 2011).

The impact of p. Arg82Trp and p. Pro186Gln mutations was slightly different upon Kir1.2 expression, although no clear distinction on cardiological phenotype was observed between the two affected patients. In this study, proband 1 with the p. Arg82Trp variant presented isolated cardiac symptoms, and proband 2 with the p. Pro186Gln variant showed extra cardiac features that allowed retrospective diagnosis of Andersen Tawil syndrome with CPVT. These data are concordant with the observation that C-terminal mutations (AA 183-428) manifested a higher prevalence of skeletal dysmorphisms than N-terminal or pore domains mutations (Manzatti et al., 2020).

It illustrates the already reported variability of expression of ATS (Tristani-Farouzi et al., 2002) and fit with the current knowledge that there is no clear genotype-phenotype correlation between *KCNJ2* variants and symptoms. Indeed, patients harboring the same variants, such as p. Arg82Trp and p. Arg82Gln can present with typical ATS, atypical ATS with cardiac features alone, isolated CPVT, or isolated LQTS (Tan et al., Kimura et al., 2012; Davies et al., 2005; Limberg et al., 2013).

To explain this variability, several hypotheses have been raised. It was suggested that N-terminal mutations could be more frequently associated with atypical ATS while C-terminal mutations with typical ATS (Kimura et al., 2012). Mutation on specific residues such as Arg67, Arg82 and Thr305 could lead to an atypical phenotype (Eckhardt et al., 2007; Kimura et al., 2012). Limberg et al. reported two *KCNJ2* mutations (p.Asn318Cys, p. Trp322Cys) associated with an isolated cardiac phenotype and the authors proposed that the mild reduction of native Kir2.1 currents due to the mutations may lead to ATS with an isolated cardiac phenotype (Limberg et al., 2013). A sex-dependent expression was described, since women appear more affected by ventricular arrhythmias whereas periodic paralysis appears more frequent in men (Andelfinger et al., 2002; Donaldson et al., 2003). Noticeably, some features of ATS can be missed, such as discrete dysmorphic features or asymptomatic arrhythmias, what happened for proband 2 for whom ATS diagnosis was retrospective. These gaps in the clinical data could contribute to apparent variability in genotype-phenotype correlation. Owing the clinical overlap between ATS and CPVT, the identification of ventricular tachycardia has to alert about neurological and morphological symptoms. Their identification would guide toward the diagnosis of ATS due to a *KCNJ2* variant, which has particular implication in the CPVT treatment strategy. On the contrary, neurological and morphological symptoms suggesting ATS should raise the hypothesis of cardiological manifestations such as CPVT.

The patients reported here were females initially referred to our laboratory through a cardiologic gateway, and diagnosed at the age of 16 and 13 with a moderate cardiologic phenotype. Interestingly, they share hallmarks of CPVT patients with *KCNJ2* mutations described so far in the literature (Table 1). *KCNJ2*-mutated patients with CPVT are all females (Andelfinger et al., 2002; Tester et al., 2006; Kukla et al., 2014), for whom the age of onset or diagnosis ranges from 2 to 36, with a mean around 15, as compared to 8 years old for *RYR2*-related CPVT (Ackerman et al., 2011). In addition, VA reported in patients with *KCNJ2* mutations have been described as milder than in *RYR2* mutated patient, especially rarely leading to syncope or sudden cardiac arrest (Andelfinger et al., 2002; Tristani-Firouzi et al., 2002; Delannoy et al., 2013; Kukla et al., 2014; Inoue et al., 2018). Reported *KCNJ2*-mutated patients with CPVT consistently show an abnormal resting ECG, while only exercise stress test triggers detectable ECG signs for other CPVT patients. The resting ECG in *KCNJ2*-mutated patients may present characteristic T-U wave patterns comprising biphasic and enlarged U-waves increasing in amplitude during tachycardia, prolonged terminal T downslope, wide T-U junctions, fusion of T and U waves after PVC, and U on P sign during sinus tachycardia (Zhang et al., 2005; Kukla et al., 2014; Inoue et al., 2018). Due to these features, QT can appear prolonged explaining why *KCNJ2*-mutated patients had long been misdiagnosed with LQTS, and ATS considered as a syndromic form of LQTS (named LQT7). In fact, appropriate measurement of the interval with tangent technique shows no QT lengthening in these patients (Zhang et al., 2005; Kukla et al., 2014). Compared to others CPVT patients, *KCNJ2-*mutated patients also show more PVC and ventricular bigeminy at rest with non-sustained and bidirectional VT (Zhang et al., 2005; Kukla et al., 2014). Noticeably, the ECG during exercise testing is also different for both groups: ventricular arrhythmias appear early, stop at the peak of exercise to finally reappear after the end of exercise for *KCNJ2*-mutated patients with CPVT, while in other CPVT patients they appear early, increase to reach the maximum at peak and stop at rest. The morphology of PVC is different between the two groups: right BBB pattern is more frequent in *KCNJ2*-mutated patients with CPVT whereas left BBB pattern if more associated with mutations in other genes (Inoue et al., 2018). Interestingly, proband 2 of our study presented a Pierre Robin sequence. Cleft palate with pathogenic *KCNJ2* mutations is rarely described in the literature (Andelfinger et al., 2002; Jakobsen et al., 2007) but it was reported that *KCNJ2* knock out homozygous mice present with a complete cleft of the secondary palate (Zaritsky et al., 2000; Dahal et al., 2012). Pierre Robin sequence or cleft palate are not specific of ATS, but our study highlights that it can represent an extracardiac sign associated with a CPVT phenotype that may evoke ATS and *KCNJ2* mutations.

**TABLE 1:** Clinical and genotype characteristics of *KCNJ2* mutation-carrying patients with ventricular arrhythmia diagnosed as CPVT in the literature.

References	Age*/Sex	Nucleotide change	Amino acid change	QTc/QUc (ms)	Abnormal U-wave	ECG abnormalities (rest/exercise)	Presentation	PP/Dysmorphy	US/MRI	Therapy	Family history
Tester et al. (2006)	*14*/F	c.244C > T	p.Arg82Trp	440/na	Yes (ns)	VE/BVT, PVT	Asymptomatic	No/No	N/na	BB	No SCD
	*9*/F	c.244C > T	p.Arg82Trp	440/na	Yes (ns)	VE/VE	Syncope-bathing (1)	No/No	N/na	BB, ICD, mexiletine	Yes (SCD)
Vega et al. (2009)	2/F	c.679G > T	p.Val227Phe	405/578	Yes (ns)	sPVC, dPVC/VE, dPVC, bPVC, BVT, nsPVT	Palpitation, exercise/emotion syncope and presyncope	No/No	Mild mitral and tricuspid impairment	BB, ICD	No
Barajas-Martinez et al. (2011)	*10*/F	c.779G > C	p.Arg260Pro	460/626	Prolonged	PVC, nsPVT, BVT/nsPVT, BVT	Palpitations, dizzinnes, syncopes	No/Yes	na	FL, (BB ineffective)	No (*de novo*)
Kimura et al. (2012)	36/F	c.431G > A	p.Gly144Asp	465/na	ns	PVC/increased PVC, PVT	Syncope, aborted CPA	No/No	na	BB, FL	No
	32/F	c.914C > G	p.Thr305Ser	443/664	ns	PVC, PVT, VF/increase PVC	Syncope, aborted CPA	No/No	na	BB, ICD	Yes
Kawamura et al., (2013)	6/F	c.431G > A	p.Gly144Asp	430/prolonged	prominent	PVC/dPVC, BVT	Exercise related syncope	No/No	na	CAT Verapamil (BB inefficient)	No SCD/CPVT
Kalscheur et al. (2014)	13/F	c.200G > A	p.Arg67Gln	430/620	Prominent	N/VE, nsPVT, BVT	Stress related syncope	No/No	N/N	CAT, BB, FL	No
Tully et al. (2015)	15/F	c.434A > G	p.Tyr145Cys	na/na	ns	na/BVT	Palpitations	No/Yes	na	ns	Yes (paternally inherited)
	10/F	c.652C > T	p.Arg218Trp	488 (then normal)/na	ns	VE, BVT, atrial ectopy	Syncope x2	Yes/Yes	na	BB, ICD	Yes (aborted CPA, SCD)
Nguyen and Ferns (2018)	9/F	c.211G > A	p.Asp71Asn	485/na	ns	VE,BVT/VE	Asymptomatic	No/Yes	N/N	BB, FL	No (*de novo*)
Current article	16/F	c.244C > T	p.Arg82trp	412/na	no	PVC, dPVC, iRBBB, atrial ectopy/bPVC, dPVC, nsPVT	Palpitations, dizziness, dyspnea, hot flushes, chest pain, asthenia	No/No	Slight dilatation of right ventricle	BB, AA	No
	13/F	c.557C > T	p.Pro186Gln	430	Yes	PVC, nsVT/dPVC, tPVC	Asymptomatic	Yes/Yes	N/N	BB, FL (poorly efficient), K^+^ supplementation	No

Abbreviations: AA, anti-arythmic; BB, beta-blockers; bPVC, bigeminy premature ventricular contractions; BVT, bidirectional ventricular tachycardia; CAT, catheter ablation therapy; CPA, cardio-pulmonary arrest; dPVC, doublet premature ventricular contractions; FL, flecainide; ICD, implantable cardioverter-defibrillator; iRBBB, incomplete right bundle branch block; N, normal; na, non-available; ns, not specified; nsPVT, non-sustained polymorphic ventricular tachycardia; PP, periodic paralysis; PVC, premature ventricular contraction; PVT, polymorphic ventricular tachycardia; SCD, sudden cardiac death; sPVC, single premature ventricular contractions; sVT, sustained ventricular tachycardia; tPVC, triplet premature ventricular contractions; US, ultrasounds; VE, ventricular ectopy; VF, ventricular fibrillation.

* Ages are indicated as age at first symptoms, otherwise they are indicated in italics

Overall, patients with clinical signs of CPVT and mutations in the *KCNJ2* gene can show a specific phenotype, which can distinguish them from individuals with CPVT associated to other genes. This is of particular importance since management can differ between both group of patients, during evaluation following the initial diagnosis, and for treatments and activity recommendations.

## Conclusion

We report here 2 novel cases of atypical ATS initially referred for suspicion of CPVT leading to the identification of *KCNJ2* pathogenic variants. We confirmed the drastic reduction of function for p. Arg82Trp mutated Kir2.1 channel and described the new loss of function p. Pro186Gln variant in *KCNJ2*. These two cases add further evidence that patients with *KCNJ2* pathogenic variants can present with cardiac arrhythmias that can phenocopy CPVT. On the bases of all published cases, it seems that patients with *KCNJ2* mutations can be identified among CPVT cohorts by accurate phenotypical description (ECG features, sex, age and severity of symptoms, associated signs). The diagnosis for these patients can however be difficult and *KCNJ2* should be included in the panel of genes used to identify molecular causes of arrhythmias, in particular to adapt CPVT patient care.

## Data Availability

The datasets for this article are not publicly available due to concerns regarding participant/patient anonymity. Requests to access the datasets should be directed to the corresponding author.
