# Supplementary figures and images for: Characterization of Loss-Of-Function KCNJ2 Mutations in Atypical Andersen Tawil Syndrome
Source: Front Genet. 2021 Nov 25;12:773177. doi: 10.3389/fgene.2021.773177 (PMC8655864; doi:10.3389/fgene.2021.773177)

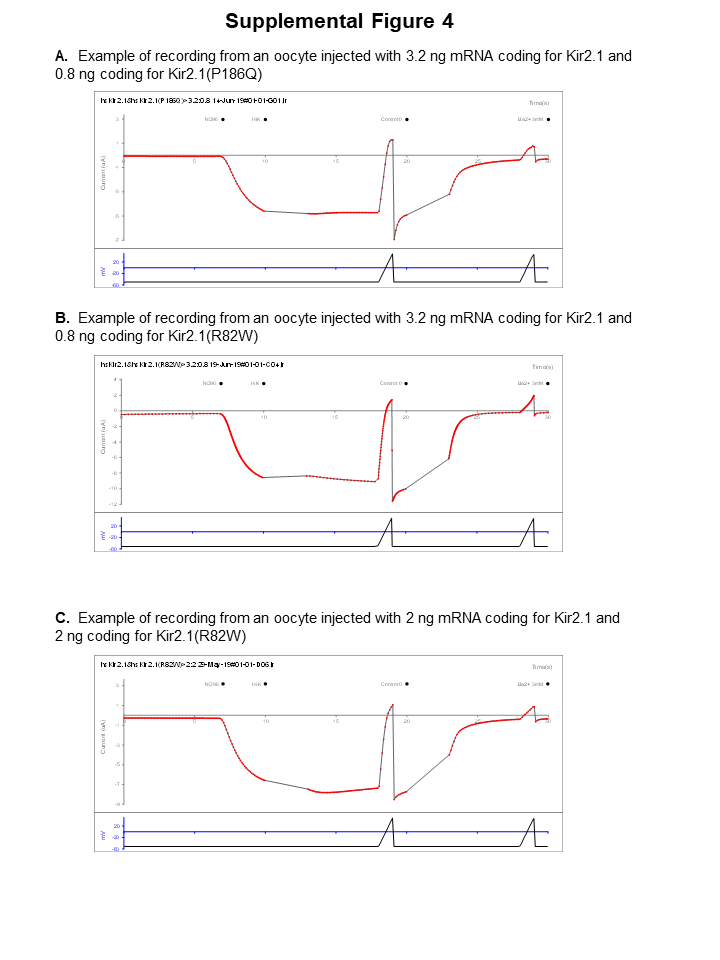

Supplement: Supplementary file 1 [file Image4.TIF]
