# Supplementary material for: Characterization of Loss-Of-Function KCNJ2 Mutations in Atypical Andersen Tawil Syndrome
Source: Front Genet. 2021 Nov 25;12:773177. doi: 10.3389/fgene.2021.773177 (PMC8655864; doi:10.3389/fgene.2021.773177)

Supplemental Figure 1

A. Protocol for measuring Kir2.1 currents with HiClamp robot

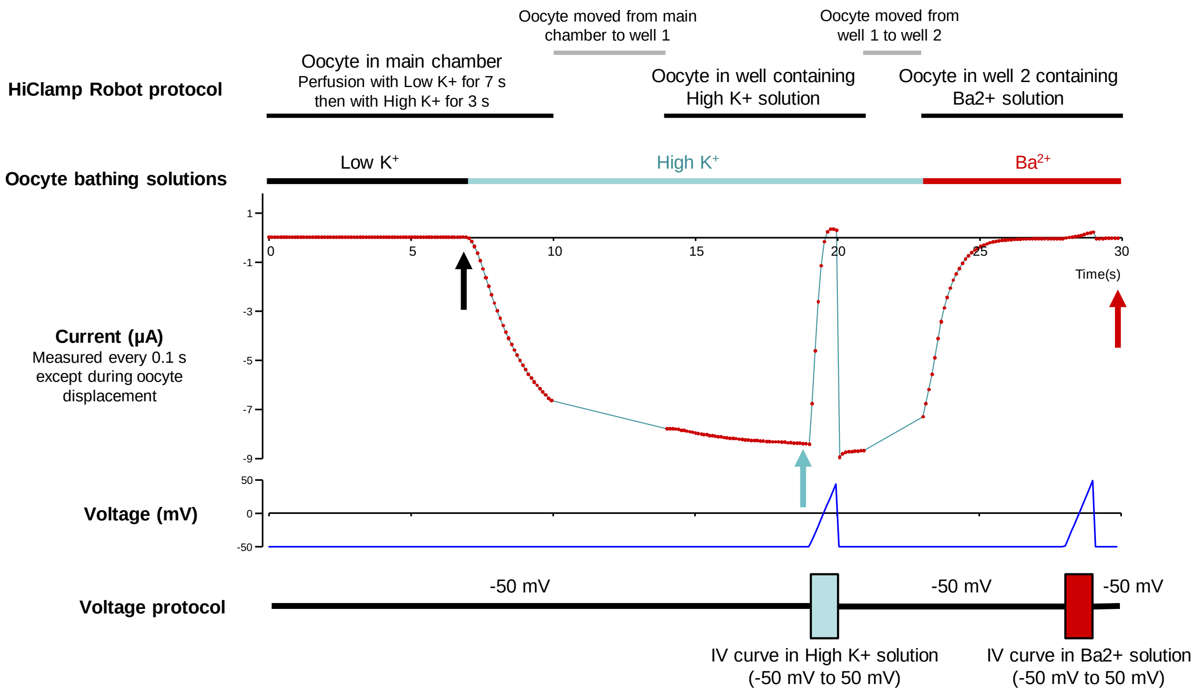

Measured values for this oocyte indicated by arrows

- Time at which Low K+ value is calculated as the average current over 0.5 s
- Time at which High K+ value is calculated as the average current over 0.5 s
- Time at which Ba2+ value is calculated as the average current over 0.5 s

Reference: Vivaudou, M., Todorov, Z., Reyes-Mejia, G. C., and Moreau, C. (2017). Ion Channels as Reporters of Membrane Receptor Function: Automated Analysis in Xenopus Oocytes. Methods Mol. Biol. 1635, 283–301

B. Actual data (WT Kir2.1) after import in Microsoft Excel and automated analysis

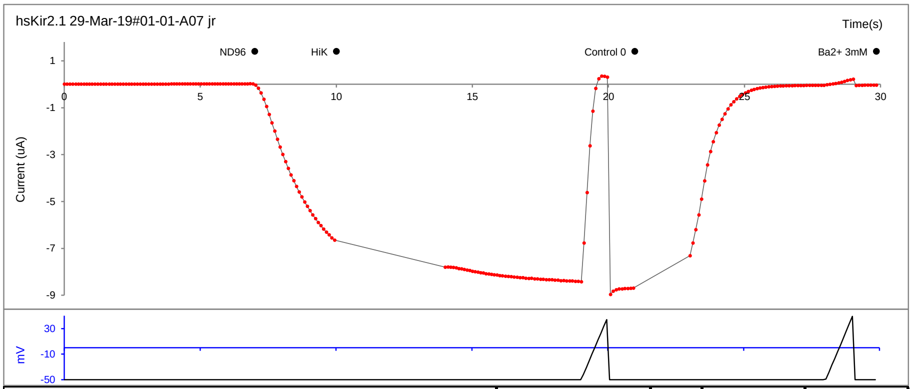

Construct	Date of experiment	Robot	Exp number	User initials
hsKir2.1	29/03/19	Yes	1-1-A07	jr

Comments

Extracted to Data_TEVC_Kir2(4/15/2019;mv)+Kir2_Data_TEVC(1/20/2021;mv)

UpperLimitYaxis | **LowerLimitYaxis** | **Quality index** 6

TimeToAverage 0.5

Solution Name	LastTimeInSolution	DeltaTime	ControlNumber	ControlToUse	I	I - Io	I - I1
				Max--->	-8.3	-8.3	-8.3
				Data->			
ND96	7	0	0	1	0.023	0	0.054
HiK	10	0		1	-6.4	-6.4	-6.4
Control 0	21	3		1	-8.3	-8.3	-8.3
Ba2+ 3mM	29.9	0	1	1	-0.031	-0.054	0

Supplemental Figure 2

A. Example of recording from an oocyte injected with 2 ng mRNA coding for Kir2.1

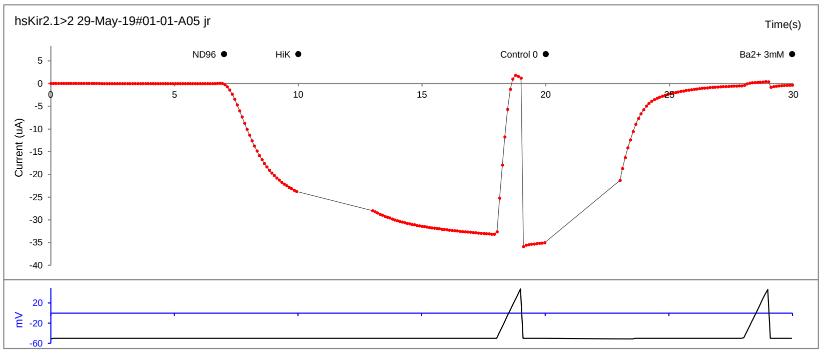

B. Example of recording from an oocyte injected with 2 ng mRNA coding for Kir2.1(P186Q)

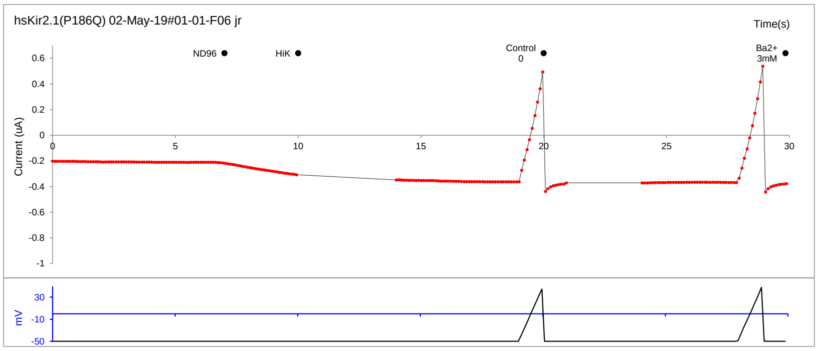

C. Example of recording from an oocyte injected with 50 ng mRNA coding for Kir2.1(P186Q)

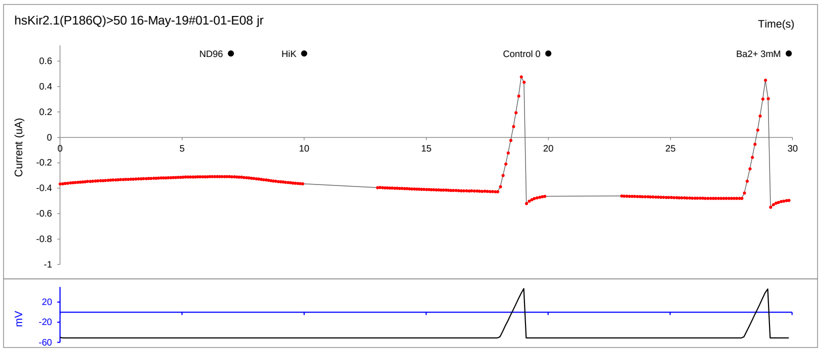

D. Example of recording from an oocyte injected with 2 ng mRNA coding for Kir2.1(R82W)
The slow opening upon change from Low K+ to High K+ was reproducible, suggesting it arises from mutation R82W but the mechanism is unknown.

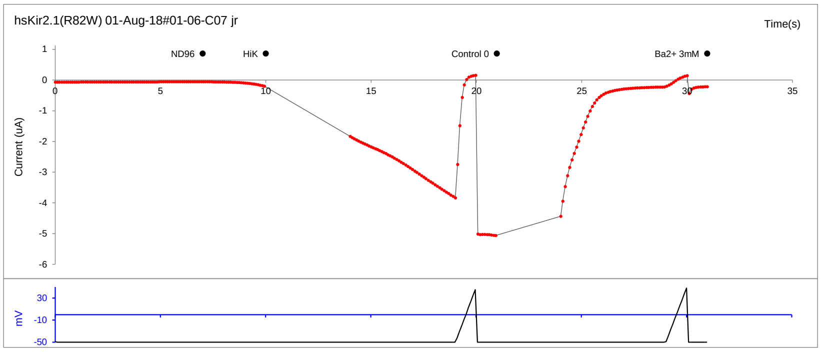

Supplemental Figure 3

A. Example of recording from an oocyte injected with 3.2 ng mRNA coding for Kir2.1 and 0.8 ng coding for Kir2.1(P186Q)

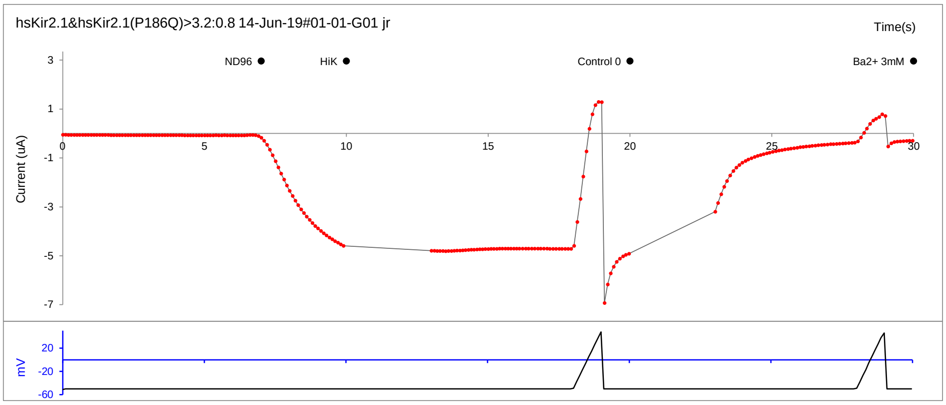

B. Example of recording from an oocyte injected with 3.2 ng mRNA coding for Kir2.1 and 0.8 ng coding for Kir2.1(R82W)

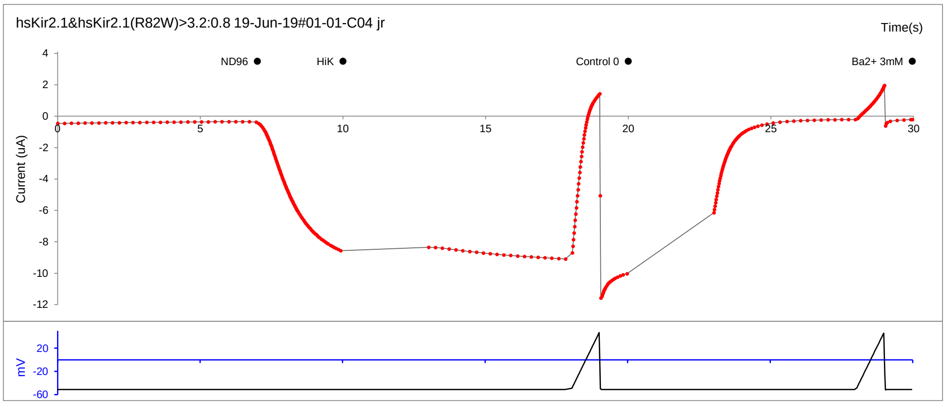

C. Example of recording from an oocyte injected with 2 ng mRNA coding for Kir2.1 and 2 ng coding for Kir2.1(R82W)

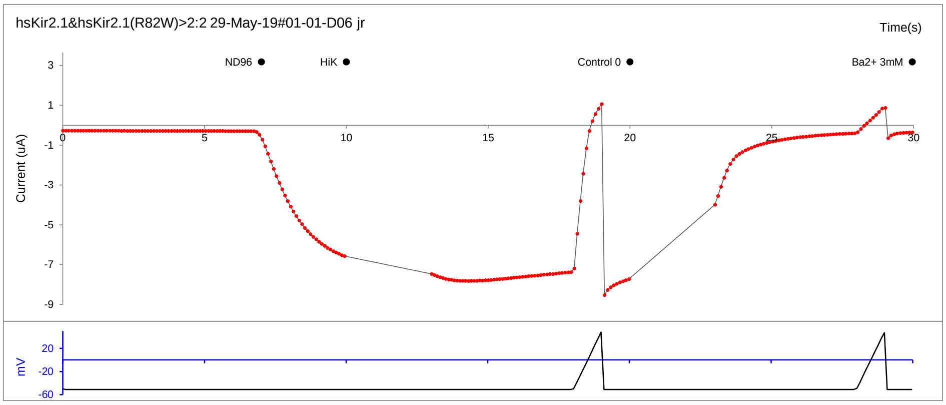

Supplement: Supplementary file 2 [file DataSheet1.pdf]
